# Tension pneumocephalus as complication of burr-hole drainage of chronic subdural hematoma: A case report

**DOI:** 10.4103/2152-7806.65185

**Published:** 2010-07-06

**Authors:** Nissar Shaikh, Irfan Masood, Yolande Hanssens, André Louon, Abdel Hafiz

**Affiliations:** 1 Department of Anesthesia/SICU, Hamad Medical Corporation, Doha, Qatar; 2 Department of Neurosurgery, Hamad Medical Corporation, Doha, Qatar; 3 Department of Clinical Pharmacy, Hamad Medical Corporation, Doha, Qatar

**Keywords:** Catheter drain insertion, Mount Fuji sign, neurological deterioration, pneumocephalus, tension pneumocephalus

## Abstract

**Background:**

Pneumocephalus is the presence of air in the cranial cavity. When this intracranial air causes increased intracranial pressure and leads to neurological deterioration, it is known as tension pneumocephalus (TP). TP can be a major life-threatening postoperative complication, especially after evacuation of chronic subdural hematoma. We report a case of TP after evacuation of chronic subdural hematoma and review the literature.

**Case Description:**

A 70-year-old man developed right-sided weakness after being admitted with minor head trauma a few weeks earlier. He was found to have a chronic subdural hematoma and underwent burr-hole evacuation. On day 3, he suddenly deteriorated and needed intubation and ventilation. Computerized tomography (CT) of the brain showed typical Mount Fuji’s sign due to TP. Immediately, 20-30 mL of air was aspirated from the intracranial fossa, and a catheter drain was inserted. The patient became fully awake after few hours and was extubated successfully. The drain was removed on day 5, and he was transferred to the ward before being discharged home.

**Conclusion:**

TP after evacuation of a chronic subdural hematoma is a neurosurgical emergency and needs immediate resuscitation and therapy; hence it is of vital importance that all acute-care physicians, intensivists and neurosurgeons be aware of this clinical emergency.

## INTRODUCTION

Presence of air in the cranial cavity is termed as *pneumocephalus*; and when this benign air causes pressure effect, it is called *tension pneumocephalus* (TP). It is manifested by neurological deterioration. TP is a rare neurosurgical emergency; if not diagnosed early and treated properly, it can be fatal. As little as 25 mL of air can cause TP.[1] TP and re-bleeding are the two major postoperative complications of evacuation of chronic subdural hematoma (CSDH).[2,6,7,9,11,16]

It is of vital importance that all acute-care physicians, intensivists and neurosurgeons be aware of this clinical entity and the need to detect it early and manage properly. We report a case of TP after evacuation of CSDH and review the literature.

## ILLUSTRATED CASE

**Figure 1:**
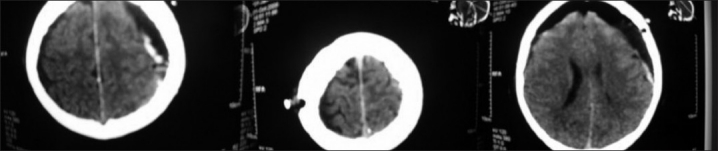
CT brain showing Mount Fuji sign

A 70-year-old man developed right-sided weakness of 3 days’ duration. He was admitted 1 month earlier with minor head trauma. The patient’s medical history included diabetes mellitus and hypertension, both controlled with medications. Computerized tomography (CT) of the head showed acute on CSDH. The same day, he underwent burr-hole evacuation of the hematoma and was postoperatively transferred to the surgical intensive care unit (SICU). He was awake and hemodynamically stable, and a follow-up CT brain evidenced pneumocephaly. On day 3, the patient became unresponsive, flexing response to deep pain, and became bradyapneic. He needed intubation and ventilation. An emergency CT evidenced TP [Figure 1]. Bedside burr-hole drainage was done. About 20 to 30 mL of air was aspirated and a catheter drain was inserted. He was nursed in supine position. He improved promptly and became awake. He was successfully weaned off the ventilator. On day 5, repeat CT revealed minimal residual air; he was extubated and the subdural drain was removed. The patient’s response improved and he remained hemodynamically stable. He was transferred to the ward on day 8 and discharged home a few days later with instructions to present for follow-up in the outpatient department.

## DISCUSSION AND REVIEW OF THE LITERATURE

Pneumocephalus is common after intracranial surgery and trauma; it usually gets absorbed without any clinical manifestations. Rarely, due to various etiological factors, this benign air will get trapped into the cranial cavity leading to an increase in the intracranial pressure and causing neurological deterioration. This clinical entity is termed *tension pneumocephalus* (TP). TP is a rare but life-threatening postoperative complication of the evacuation of CSDH.[2,3,5,7,11,14,16]

### Epidemiology and risk factors

So far, only 30 cases of TP following the evacuation of CSDH have been reported [Table 1], with a reported incidence of TP varying from 2.5% to 16%.[3,8,16]

**Table 1:** Tension pneumocephalus after evacuation of chronic subdural hematoma — number of cases and treatment

Author	Primary Etiology	Number of Cases	Treatment Given
Sharma *et al*.[16]	Chronic subdural hematoma (CSDH)	5	Twisted-drill craniostomy and aspiration of air
Cummins[7]	CSDH	1	Catheter aspiration of air
Mori and Maeda[14]	CSDH	4	Reopening the wound
Parkash *et al*.[15]	CSDH	1	Invasive ventilation
Toung *et al*.[19]	CSDH	4	Craniostomy
Kawakami *et al*.[10]	CSDH	1	Closed-system drain
Bremer and Nguyen[3]	CSDH	3	Craniostomy and drain
Caron *et al*.[5]	CSDH	1	Craniostomy and lumbar subarachnoid infusion
Bouzarth *et al*.[4]	CSDH	5	Percutaneous catheter drain with –ve pressure
Ishiwata *et al*.[8]	CSDH	5	Burr hole

The main risk factors are (i) replacement of chronic subdural hematoma with oxygen,[1] (ii) position and duration of the surgery,[16,17] (iii) intraoperative administration of mannitol or frusemide,[7,16,17] (iv) nitrous oxide anesthesia,[9] (v) gross hydrocephalus,[13] (vi) functioning ventriculo-peritoneal shunt,[13,17] (vii) lumbo-peritoneal drainage[12] and (viii) drain insertion after the hematoma cavity is irrigated.[11]

### Physiopathology

TP occurs when the volume of cerebrospinal fluid (CSF) decreases during surgery, but it can also occur due to other reasons. Air will rush into the brain spaces via bony or dural defects to fill, and get collected in, the negative pressure space created by the loss of CSF. When the CSF volume is restored, but volume of air remains the same, it leads to air trapping and TP.[16]

### Diagnosis

A CT scan of the brain is the most sensitive method and a gold standard for diagnosis of TP. CT imaging is also helpful in differentiating TP from benign forms. The CT will show the typical ‘Mount Fuji’, sign – bilateral hypo-attenuating collection causing compression and separation of the frontal lobes and widening of interhemispheric space between the tips of the frontal lobe, giving a picture of the silhouette of Mount Fuji [Figure 1].[16]

Criteria for the diagnosis of TP are (a) typical CT brain findings, (b) neurological deterioration, (c) hissing sound of escape of air and (d) immediate improvement in the neurological status upon aspiration of air.[16] An exception to these criteria has recently been reported, that even smaller amounts of air around the brainstem can lead to compression of the vital center, causing neurological and respiratory deterioration.[15]

### Management

TP is a neurosurgical emergency, and the patient should immediately be started on a higher concentration of inspired oxygen and maintained in supine position. If the Glasgow coma score (GCS) decreases below 8, endotracheal intubation is needed. Simultaneously, one can reopen the frontal burr hole to release the air; or a new bedside burr hole can be made for aspiration of the air and insertion of a catheter drain to lessen the risk of infection; or a twisted-drill craniotomy can be performed with insertion of a drain.[2,7,16]

In patients with evacuation of CSDH, brain tissues will not expand immediately to fill the space created. To avoid development of TP, flushing of subdural space with saline, placement of patient on 100% oxygen before closing the wound with head elevation in supine position, and insertion of close-system drain should be considered. The drain can be kept for a maximum of 48 hours to minimize the chance of infection, venting the residual intracranial air, and the patient should be nursed in a supine position.[2] Burr-hole aspiration of subdural hematoma, without drain insertion, can cause sudden decompression of the brain and may lead to convulsion or postoperative brain infarction.[3] Aung *et al*. inserted a closed-system drain in all patients with post-’chronic subdural hematoma evacuation’, and none of their patients had TP or brain infarction or convulsion in the postoperative period.[2]

Sharma *et al*.[16] recommended twisted-drill craniotomy and aspiration of the air using a brain cannula with three-way connection. Caron *et al*.[5] managed their case of TP after evacuation of CSDH by craniotomy drainage and with continuous lumbar subarachnoid infusion. While Toung *et al*.[19] treated their TP patient by craniostomy, Bouzarth[4] described percutaneous catheter connected to negative pressure for release of TP. Cummins used indwelling drain catheter to aspirate the air and relieve TP in his patient.[7] An overview of reported cases is summarized in Table 1.

There are advantages and disadvantages [Table 2] of keeping the drain after evacuation of CSDH. The benefits of inserting the drain are a significant decrease in the incidence of recurrence of hematoma; and re-evacuation of hematoma, as well as continuous drainage of residual hematoma and pneumocephalus.[20] Drains also decrease the convalescent period and promote wound healing.[7] The disadvantages are brain tissue injury,[6] risk for infection, subdural empyema and bleeding.[18]

**Table 2:** Pros and cons of drain, after evacuation of chronic subdural hematoma

Pros	Cons
Significantly prevents recurrence of hematoma[20]	Brain injury[6]
Decreases re-operation rate[20]	Infection[18]
Drains residual hematoma[20]	Subdural empyema[18]
Promotes wound healing[6]	Hemorrhage from neomembrane[18]
Decreases convalescent period[6]	‐

We believe that, keeping the drain helps the patient, and disadvantages of the drain can be prevented by meticulous precautions during insertion, following strict aseptic technique and removal of the drain as early as possible.

Rarely, a smaller amount of air in the extra-axial space around the brainstem after evacuation of CSDH in supine position can cause vital center compression, as reported by Parkash *et al*.[15] They reported that, this led to localized tension. Due to the location and tiny pneumocephalus, this was managed conservatively. The patient improved after a few hours of invasive mechanical ventilation.[15]

### Prevention

Prevention of TP begins as soon as the patient gets admitted to the hospital with the primary pathology and includes obtaining consent for the surgical procedure, educating the patient and family about the risk factors of TP and applying all possible measures to avoid them. Intraoperative saline flushing, avoiding nitrous oxide, supine position and a closed-system drain will all help in preventing TP.[2,16,20,21] Kravtchouk *et al*.[11] suggested that rapid manipulation associated with insertion and removal of the drain, keeping the drain sealed during the entire procedure, may prevent occurrence of TP. Finally, meticulous postoperative neurological monitoring is vital for early detection of any deterioration.

## CONCLUSION

TP after evacuation of the chronic subdural hematoma is a neurosurgical emergency. Closed-system drains will significantly prevent TP. TP needs immediate resuscitation and therapy; hence it is of vital importance that all acute-care physicians, intensivists and neurosurgeons be aware of this clinical emergency.
